# Mitigating Health-Related Uncertainties During Pregnancy: The Role of Smart Health Monitoring Technologies

**DOI:** 10.2196/48493

**Published:** 2024-03-25

**Authors:** Max Tretter

**Affiliations:** 1 Chair of Systematic Theology (Ethics) Seminar for Systematic Theology Friedrich-Alexander-Universität Erlangen-Nürnberg Erlangen Germany

**Keywords:** stress, anxiety, reproductive technologies, fetal health, epistemology, ethics

## Abstract

Pregnancy is a time filled with uncertainties, which can be challenging and lead to fear or anxiety for expectant parents. Health monitoring technologies that allow monitoring of the vital signs of both the mother and fetus offer a way to address health-related uncertainties. But are smart health monitoring technologies (SHMTs) actually an effective means to reduce uncertainties during pregnancy, or do they have the opposite effect? Using conceptual reasoning and phenomenological approaches grounded in existing literature, this Viewpoint explores the effects of SHMTs on health-related uncertainties during pregnancy. The argument posits that while SHMTs can alleviate some health-related uncertainties, they may also create new ones. This is particularly the case when the abundance of vital data overwhelms pregnant persons, leads to false-positive diagnoses, or raises concerns about the accuracy and analysis of data. Consequently, it is concluded that the use of SHMTs is not a cure-all for overcoming health-related uncertainties during pregnancy. Since the use of such monitoring technologies can introduce new uncertainties, it is important to carefully consider where and for what purpose they are used, use them sparingly, and promote a pragmatic approach to uncertainties.Using conceptual reasoning and phenomenological approaches grounded in existing literature, the effects of SHMTs on health-related uncertainties during pregnancy are explored. The argument posits that while SHMTs can alleviate some health-related uncertainties, they may also create new ones. This is particularly the case when the abundance of vital data overwhelms pregnant persons, leads to false-positive diagnoses, or raises concerns about the accuracy and analysis of data. Consequently, it is concluded that the use of SHMTs is not a cure-all for overcoming health-related uncertainties during pregnancy. Since the use of such monitoring technologies can introduce new uncertainties, it is important to carefully consider where and for what purpose they are used, use them sparingly, and promote a pragmatic approach to uncertainties.

## Introduction

Pregnancy is a time full of uncertainties [1], starting from uncertainties concerning the health of the fetus and pregnant person, to parental uncertainties about how life will change during pregnancy and after birth, to socioeconomic uncertainties [2,3]. These uncertainties can be very challenging and can cause fear and anxiety among those affected [4]. Smart health monitoring technologies (SHMTs) offer the possibility to continuously monitor the health of both the child and the mother, allowing parents to at least mitigate some health-related uncertainties, thereby alleviating associated stress and anxiety.

But are SHMTs actually an effective means to reduce uncertainties during pregnancy, or do they have the opposite effect? In this Viewpoint, I will explore this question at a conceptual level and argue that while SHMTs can indeed reduce some health uncertainties, their use may also introduce new ones.

The argument is developed as follows: First, I define the concept of uncertainty, drawing on relevant theories of uncertainty. Then, using conceptual reasoning supported by existing research, I theorize how pregnancy screenings can address certain uncertainties and how SHMTs can alleviate the remaining ones. Following this, I adopt a phenomenological approach to examine how the introduction of these technologies could create new uncertainties. This analysis is underpinned by key studies identified from database searches on PubMed, Embase, Web of Science, and Google Scholar using the keywords “pregnancy” and “uncertainty.” Finally, I reflect on the implications and limitations of these insights and provide practical recommendations based on pragmatic considerations.

## Uncertainties in the Context of Pregnancy

Both in common sense and in the scientific discussion of this concept, uncertainty is largely understood as an epistemic state [5,6] in which individuals lack knowledge because they either lack information or the available information is inaccurate or incorrect [7]. Many people experience uncertainty as a challenge, which can lead to stress, anxiety, and fear [8]. A well-established method to navigate these uncertainties and alleviate the ensuing negative emotions involves bridging these knowledge gaps [9].

In the realm of health, uncertainty is nearly ubiquitous [10-12]. Even among those with a solid grasp of their bodily functions and biological processes, there remain gaps in knowledge and looming questions: What will my health look like tomorrow, next week, or a year from now [13]? What steps can I take to prolong my well-being? The same can be said for pregnancy [14], as pregnant women undergo significant physical and psychological changes and are constantly confronted with uncertainty [15] regarding whether the fetus is developing healthily, whether there are signs of potential health issues, whether they are behaving correctly, or whether they need to make changes to promote the healthy development of the fetus [3]. Pregnancy, an inherently stressful period, can be further complicated by these uncertainties, potentially escalating stress and anxiety levels in expectant mothers [15], which may adversely affect both the parent and child [16,17]. Therefore, it is crucial to mitigate these uncertainties where feasible, to minimize unnecessary stressors.

## Pregnancy Screenings to Address Health-Related Uncertainties

Thankfully, expectant parents are not helpless in the face of these health-related uncertainties. Various prenatal tests and screenings routinely conducted throughout pregnancy can provide insight into the health of both the pregnant individual and the fetus, thereby helping to alleviate existing uncertainties.

Pregnancy screenings typically involve monitoring the vital signs of the pregnant person, including blood pressure, heart rate, and weight [18]. By tracking weight gain, health care providers can assess the health of both the pregnant person and fetus, as inadequate weight gain may be harmful to the fetus, while rapid or excessive weight gain may be a sign of health concerns [19,20], such as gestational diabetes [21,22] or pre-eclampsia [23]. A pregnant person’s heart rate and blood pressure can also provide valuable information, with a low maternal heart rate potentially negatively affecting fetal growth and birth weight of the fetus [24,25], while high blood pressure or an abnormal heart rate is associated with increased risks of gestational diabetes [26] and maybe a sign of future cardiovascular disease [27,28]. Monitoring these vital signs is crucial for safeguarding the health of both the pregnant person and fetus, enabling timely interventions if necessary, and ultimately reducing health-related uncertainties during pregnancy.

## Smart Health Monitoring Technologies to Address These Uncertainties

Although pregnancy screenings are vital for ensuring the health of both the pregnant person and the fetus, weeks can pass between appointments. During this time, pregnant individuals may find themselves grappling with everyday uncertainties, such as the effects of dietary changes or physical activities on the fetus, and seeking reassurance about both their own well-being and that of their unborn child.

SHMTs such as smartwatches, smart rings, and fitness trackers, especially when integrated within an Internet of Things ecosystem [29,30], can offer a practical solution [31-33]. These devices enable parents to independently measure the vital signs of the pregnant person and, in some cases, the fetus at home. This data can be analyzed to provide parents with an assessment of their fetus’s health condition without needing to visit a doctor. The capability to ascertain the health status of their fetus from the comfort of home, or to receive timely alerts if the data points to potential health issues, could provide much-needed reassurance in between medical screenings and counter emerging uncertainties.

## New Uncertainties Produced by the Use of Smart Health Monitoring Technologies

The widespread acceptance of SHMTs among expectant parents [34], coupled with the fact that their use is considered extremely feasible by them [35], as well as the empirical evidence suggesting that their use can have a positive impact on the health of pregnant persons [36] and can reduce their stress levels [37], should not obscure the fact that their use also brings some disadvantages. In addition to privacy and data protection issues [38,39], the use of SHMTs can also produce new health-related uncertainties.

First, new information can in itself produce new uncertainties [40], particularly when there are no medical personnel available to help interpret the additional data. In the absence of such guidance, every recorded irregularity, whether it be an irregular heartbeat, temporary high blood pressure, or a decrease in blood oxygen saturation, can cause pregnant persons to question their own health and that of their child. In extreme cases, these uncertainties can lead to unnecessary anxiety and stress symptoms [41,42], which can be detrimental to their mental and emotional well-being.

However, even in professional contexts, more data might produce new uncertainties. This happens, for example, when continuous health monitoring triggers false alarms [43], as, for instance, particular vital sign patterns are erroneously interpreted as symptoms of a specific disease or indicators of an emergency, despite being totally harmless. As Welch et al explain in their publication *Overdiagnosed* [44], the likelihood of such false-positive diagnoses inevitably increases with additional data collection, which can lead to unnecessary or even incorrect diagnoses and create additional health uncertainties.

Last, there is the uncertainty of whether health data are being collected accurately and analyzed correctly. Users of SHMTs must ask themselves if the sensors are properly attached, if smart devices are being used correctly, and if the data are being transmitted and analyzed correctly by the app’s algorithms [45]. Particularly for medical and technical “laypeople,” there is no way to verify this, and they must live with the uncertainty about the reliability of the data.

## Ambivalent Effects of Pregnancy Monitoring Technologies

I have shown how SHMTs can mitigate the daily health uncertainties encountered during pregnancy. However, it is important to note that the use of SHMTs during pregnancy can also create new uncertainties as they make even the slightest abnormalities visible, increase the likelihood of false-positive results, and raise constant questions about the reliability of the data.

In summary, the use of SHMTs should be viewed as a double-edged sword. While they can help alleviate health uncertainties and reassure and relax pregnant persons, they also carry the risk of introducing new uncertainties, potentially exacerbating stress, anxiety, and fear.

## Limitations and Opportunities for Further Research

The discussion regarding the correlation between SHMTs and uncertainties during pregnancy highlights 2 significant limitations. While these limitations require careful consideration, they do not invalidate the fundamental insights. Rather, they should be interpreted as avenues for further investigation.

First, I did not present original data to substantiate my arguments. Although I have incorporated recent literature to substantiate key assumptions, and empirical research on uncertainty in pregnancy [46] and on the impact of (health) technologies on uncertainty [47] support my findings, my contribution was strictly conceptual.

Second, the results do not explore the balance between the emergence of new uncertainties and the resolution of preexisting ones. Does the use of SHMTs produce more health uncertainties than it reduces, or vice versa? This question cannot be answered definitively, as shifts in uncertainty resulting from the use of such technologies need to be assessed on a case-by-case basis.

## Practical Recommendations

In conclusion, the question arises of what practical recommendations can be derived from the insights gained regarding the use of SHMTs during pregnancy. Considering the preceding discussions, I recommend not to use SHMTs in terms of “the more the better,” as their increased use might also produce more uncertainties. Instead, it seems advisable to use them sparingly and only where constant monitoring is actually necessary [10], such as when abnormalities have been identified during pregnancy screening or when there are hereditary medical conditions. In cases where constant monitoring is not necessary, these technologies should only be used upon explicit request from parents and with appropriate training on their use and data interpretation.

Moreover, even if it may sound cynical at first, a pragmatic approach toward uncertainties in health care (not just during pregnancy) can be helpful and should be encouraged [11,48-51]. This involves acknowledging that not everything can be controlled and that sometimes it can be beneficial to simply let things “take their course” [52,53].

## Abbreviations

SHMTs: smart health monitoring technologies
